# Prevalence and clinical consequences of Hepatitis C virus infection in patients undergoing hematopoietic stem cell transplantation

**DOI:** 10.1590/S1678-9946202466011

**Published:** 2024-02-05

**Authors:** Ana Claudia Marques Barbosa Diaz, Steven Sol Witkin, Cesar de Almeida, Alfredo Mendrone, Vanderson Rocha, Silvia Figueiredo Costa, Jessica Fernandes Ramos, Maria Cassia Mendes-Correa

**Affiliations:** 1 Universidade de São Paulo, Faculdade de Medicina, Departamento de Moléstias Infecciosas e Parasitárias, São Paulo, São Paulo, Brazil; 2 Universidade de São Paulo, Faculdade de Medicina, Instituto de Medicina Tropical de São Paulo, Laboratório de Investigação Médica em Virologia (LIM-52), São Paulo, São Paulo, Brazil; 3 Weill Cornel Medicine, Department of Obstetrics and Gynecology New York, New York, USA; 4 Fundação Pró-Sangue, Hemocentro de São Paulo, São Paulo, São Paulo, Brazil; 5 Universidade de São Paulo, Faculdade de Medicina, Disciplina de Ciências Médicas, São Paulo, São Paulo, Brazil; 6 Universidade de São Paulo, Faculdade de Medicina, Hospital das Clínicas, Departamento de Hematologia, Laboratório de Investigação Médica em Patogênese e Terapia Dirigida em Onco-Imuno-Hematologia (LIM-31), São Paulo, São Paulo, Brazil; 7 Universidade de São Paulo, Faculdade de Medicina, Instituto de Medicina Tropical de São Paulo, Laboratorio de Investigação Médica em Virologia (LIM-49, São Paulo, São Paulo, Brazil; 8 Universidade de São Paulo, Faculdade de Medicina, Hospital das Clínicas, São Paulo, São Paulo, Brazil

**Keywords:** Hepatitis C, Hematopoietic stem cell transplantation, Prevalence, Clinical outcome

## Abstract

Hepatitis C virus (HCV) infection is a significant cause of morbidity and mortality among hematopoietic stem cell transplant (HCT) recipients. In Brazil, its occurrence in HCT recipients remains undetermined. We now report on HCV prevalence in HCT recipients and its clinical consequences. The medical records of all HCT recipients seen at Hospital das Clinicas, Sao Paulo University Medical School, from January 2010 to January 2020 were reviewed to determine HCV serostatus. A retrospective analysis of medical charts was undertaken on all seropositive cases to determine HCV genotype, presence of liver fibrosis, co-infections with other viruses, previous treatments, and clinical evolution of liver pathology after HCT. Of the 1,293 HCT recipients included in the study, seven (0.54%) were HCV antibody-positive and five (0.39%) were also viremic for HCV-RNA. Four of these individuals had moderate to severe liver fibrosis (METAVIR F2/F3) and one was cirrhotic. Two of the viremic patients developed acute liver dysfunction following transplantation. All patients had their acute episode of liver dysfunction resolved with no further complications. Four of the viremic patients were treated for HCV infection with direct acting agents (DAA). Information regarding HCV treatment was lacking for one of the viremic HCV patients due to loss of follow up. Sustained anti-virologic responses were observed in three cases after the use of DAA. The detection of HCV in hematological adults undergoing HCT and its successful treatment with DAA highlight the necessity of testing for HCV both prior to and following transplantation.

## INTRODUCTION

Hepatitis C is a worldwide concern. Currently, it is estimated that worldwide 58 million people have chronic infection with hepatitis C virus (HCV), with 1.5 million new cases of the disease developing annually^1^. In Brazil, number of HCV-seropositive individuals was estimated at 0.53% in 2017 and the number of viremic individuals positive for HCV RNA at 632,000, corresponding to 0.31% of the population aged 15 to 69 years old^2^.

Hematopoietic stem cell transplant (HCT) recipients with chronic HCV infection, untreated for this infection, are at elevated risk of complications following transplantation due to viral reactivation or exacerbation. This includes sinusoidal obstruction syndrome, liver dysfunction, and increased mortality^3-9^. One-third of HCV-infected HCT recipients subsequently develop end-stage liver disease (cirrhosis, hepatocellular carcinoma, or disease requiring liver transplantation). Fatal fibrosing cholestatic disease has also been reported^3^. The HCV is commonly associated with extra-hepatic manifestations including hematologic malignancies, particularly Non-Hodgkin’s Lymphoma (NHL). The low cumulative incidence of NHL upon eradication of HCV, as well as the frequent recurrence of the neoplasm after viral recurrence, corroborate this association^10,11^.

For many years, interferon (IFN)-based treatment has been the standard therapy for HCV infection. However, this regimen is associated with poor tolerability, low efficacy, and significant adverse side effects, including graft failure and graft-versus-host disease (GVHD), anemia, and neutropenia^12-14^. Current guidelines recommend that, when possible, all HCV-infected HCT candidates should start therapy and should complete therapy for HCV before transplant^3^. After HCT, HCV-infected patients who develop fibrosing cholestatic hepatitis C, patients with cirrhosis whose condition is deteriorating, and patients who underwent HCT for HCV-related lymphoproliferative disorders should be treated for HCV without delay. Additionally, according to current guidelines all HCV-infected long-term survivors of HCT should be offered antiviral therapy^3^. However, little is known about the impact of treatment with these new drugs on the clinical course of HCV infection after transplantation^15-19^. In Brazil, data on HCV infection among HCT recipients is very scarce.

This exploratory study aimed to evaluate the prevalence of HCV infection and its impact on clinical outcome in patients undergoing HCT.

## MATERIALS AND METHODS

### Study design and study population

We performed an observational, retrospective, single-center study at the Division of Hematology (Fundacao Pro-Sangue/Hemocentro de Sao Paulo) at Hospital das Clinicas, Sao Paulo University Medical School. This service is a national reference in the area and participates in the Brazilian Bone Marrow Transplantation Program, accounting for more than 3,000 predominantly autologous transplants among adult patients accomplished since its foundation in 1988.

The medical records of all HCT recipients treated at the institution, from January 2010 to January 2020, were reviewed to determine HCV serological status. Inclusion criterion was the availability of information on HCV serologic status before HCT. After identifying the seropositive cases, a retrospective chart analysis was conducted to evaluate patient demographics, HCV genotype, stage of liver fibrosis, co-infections with other viruses, history of previous treatment as well as the type of viral response observed and the occurrence of serious adverse events during and following the transplant.

Prior to their HCT, all recipients were routinely screened for HIV, Hepatitis B surface antigen (HBsAg), anti-HBc, HCV, human T-cell lymphotropic virus (HTLV), syphilis, and Chagas disease by serology. Hepatitis B virus, HIV, and HCV RNA were also tested by nucleic acid detection in all samples after 2014. Syphilis and Chagas disease testing are mandatory before any HCT according to Brazilian federal regulations, due to their high endemic occurrence. The HCV infection was defined as the presence of detectable anti-HCV antibody or HCV RNA in serum prior to HCT.

Liver fibrosis, including cirrhosis was diagnosed via liver biopsy or a combination of noninvasive fibrosis markers (liver elastography, APRI, and FIB four scores), radiologic findings, and clinical manifestations of cirrhosis. The HCV reactivation was defined as an increase in the HCV RNA viral load of at least 1 log_10_ IU/mL over baseline after chemotherapy or immunosuppressive therapy^3^.

Acute exacerbation of chronic HCV infection was defined as a three-fold or greater increase in the serum ALT level above baseline in the absence of cancerous liver infiltration; use of hepatotoxic medications; blood transfusion within one month of elevation of ALT level; or other systemic infections affecting the liver (including hepatitis A virus, hepatitis B virus, cytomegalovirus, adenovirus, herpes simplex virus, varicella-zoster virus, and HIV infections)^3^.

Sinusoidal obstruction syndrome (SOS) had a clinical diagnosis based on the Seattle or Baltimore criteria, which included the presence of at least two of the following symptoms up to 30 days post-HCT: jaundice, hepatomegaly and right upper quadrant pain, and ascites and/or unexplained weight gain (≥ 2%). The modified Seattle criteria include the presence of at least two of the following symptoms up to 20 days post-HCT: hyperbilirubinemia ≥ 2 mg/dL, hepatomegaly or right upper quadrant pain of hepatic origin, and sudden weight gain (≥ 2%). The Baltimore criteria include hyperbilirubinemia ≥ 2 mg/dL up to 21 days post-HCT and at least two of the following symptoms: hepatomegaly, ascites, and weight gain (≥ 5%)^8^.

### HCV RNA quantification and genotyping

The HCV RNA level was measured by a commercial real-time polymerase chain reaction (PCR) with a detection limit of 12 IU/mL (Abbott Molecular, Des Plaines, IL). The HCV genotype was determined using direct sequence analysis of the 5’ noncoding region with the VERSANT HCV Genotype 2.0 assay (Siemens Healthcare Diagnostics Division, Tarrytown, NY). The HCV RNA levels were measured at the beginning of treatment and at weeks 12 and/or 24 after the end of therapy.

### HCV treatment

The decision to initiate treatment and the choice of which direct acting agents (DAA) was entirely at the discretion of the attending physician and in accordance with guidelines of the Brazilian Ministry of Health and the product manufacturers. Effective HCV treatment was defined as a negative viral load for at least 12 and/or 24 weeks following the discontinuation of anti-viral therapy (sustained anti-virologic response). The severity of adverse events was graded with the Division of AIDS Grading Table (version 1.0, December 2004; clarification, August 2009)^20^.

### Clinical evolution and follow-up

Clinical outcome was assessed by consulting medical records. Transplant variables consisted of HCT type and age at transplant. In HCV-infected recipients, toxicity during HCV treatment and liver disease progression were determined by analyzing laboratory tests and clinical evolution for at least a year after HCV treatment or HCT, respectively. Laboratory tests included: creatinine clearance (CKD-EPI); alanine aminotransferase (ALT); aspartate aminotransferase (AST); alkaline phosphatase (AP); gamma-glutamyl transferase (GGT); direct bilirubin (DB); indirect bilirubin (IB); albumin (Alb); prothrombin time (PT/INR); neutrophil; hemoglobin and platelet count; and HCV RNA.

### Ethical aspects

The study was approved by the local ethics committee, CAPPesq – Comissao de Etica para Analise de Projetos de Pesquisa do HCFMUSP, protocol Nº CAAE: 12151619.7.0000.0068. Since all subjects were de-identified, informed written consent was deemed unnecessary.

### Statistical analysis

Descriptive statistics were used to summarize the patients’ demographic, clinical, treatment, and outcome variables. Categorical variables were presented as frequencies and percentages, and continuous variables were presented as median and ranges of values.

## RESULTS

### Patient characteristics

**Figure 1:**
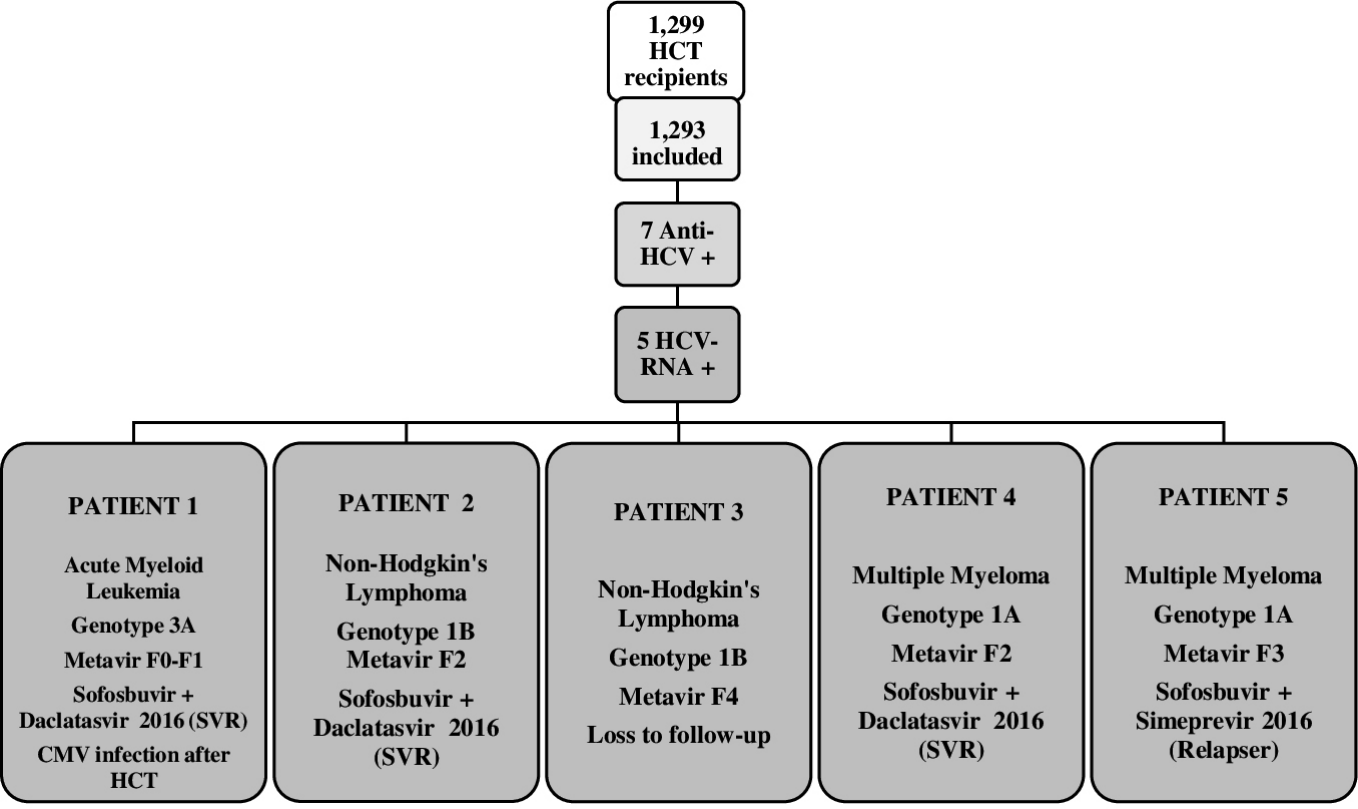
- Summary of the selection, inclusion and clinical and therapeutic findings of the hematopoietic stem cell transplant recipients. CMV = cytomegalovirus; SVR = sustained virological response; HCT = hematopoietic stem cell transplant.

Among 1,299 hematopoietic stem cell transplant recipients seen during the study period, 1,293 met the inclusion criterion. Seven individuals were anti-HCV antibody positive, five of whom were also positive for HCV-RNA (Figure 1). Table 1 and Figure 1 shows their characteristics. Their median age at the time of transplantation was 51.3 years (range 31–64 years), most (five) were male. It was not possible to determine the duration of their HCV infection or the mode of HCV acquisition due to incomplete clinical data in the medical charts. Among the seven HCV-antibody positive patients, the reasons for HCT were diffuse large B-cell lymphoma (two cases), mantle cell lymphoma (two cases), and multiple myeloma (two cases), acute myeloid leukemia (one case).

**Table 1:** - Characteristics of the HCV antibody positive hematopoietic stem cell transplant recipients.

General characteristics	N = 7 (%)
Male sex	5 (71.4)
Median age in years (range)	53.1 (31–64)
Type of HCT	
Autologous	6 (85.7)
Allogeneic	1 (14.3)
Hematological disease	
Acute Myeloid Leukemia	1 (14.3)
Diffuse large B cell lymphoma	2 (28.6)
Mantle Cell lymphoma	2 (28.6)
Multiple Myeloma	2 (28.6)
Antibody positive	
HBsAg	0
Anti-HBc	0
Anti-HBs	5 (71.4)
Anti-HIV	0
Anti-HTLV 1 / 2	1 (14.3)
Syphilis	0
Chagas disease	0
HCV RNA positive	5 (71.4)

Table 2 shows the five HCV-RNA positive patients. Four were positive for HCV genotype 1 and had moderate to severe liver fibrosis (two METAVIR F2, one METAVIR F3, one METAVIR F4). One patient had established cirrhosis at baseline with compensated liver disease (Child-Pugh class A) and for this patient hepatitis C genotype was not available.

**Table 2:** - Characteristics the HCV-RNA positive Hematopoietic Stem Cell Transplant recipients.

General characteristics	N = 5 (%)
Male sex	4 (80)
Median age in years (range)	51.8 (31–56)
Type of HCT	
Autologous	4 (80)
Allogeneic	1 (20)
Hematological disease	
Acute Myeloid Leukemia	1 (20)
Diffuse large B cell lymphoma	1 (20)
Mantle Cell lymphoma	1 (20)
Multiple Myeloma	2 (40)
Antibody positive	
Anti-HBs	5 (100)
Anti-HTLV 1 / 2	1 (20)
HCV genotype 1	4 (80)
METAVIR ≥ F2	4 (80)
Liver cirrhosis	1 (20)
HCV therapy	4 (80)
Post HCT liver dysfunction	2 (40)
CMV	1 (20)
SOS	1 (20)
Liver complications	0
Clinical outcome	
Follow up	4 (75%)
Lost to follow up	1 (25%)
Death	0

CMV = cytomegalovirus; SOS = hepatic sinusoidal obstruction syndrome.

### Safety and efficacy of HCV treatment

Among the five viremic patients, four received a total of nine antiviral treatment attempts, either before (five) or after (four) HCT. Information regarding HCV treatment was absent for one of the viremic HCV patients, due to loss of follow up. All patients who received IFN-based therapy did so before transplantation. Treatment involved either conventional or pegylated IFN with ribavirin.

All patients who received pre-HCT IFN-based regimens had grade 3 adverse events. Among them, 75% developed anemia and neutropenia and 25% developed thrombocytopenia. Only one case of antiviral treatment discontinuation was reported (grade 4 adverse event). No sustained virologic response was observed for any of these treatments (Table 2). All four patients received DAA after their unsuccessful IFN-based treatments. Two patients received DAA (Sofosbuvir-Simeprevir or Sofosbuvir-Daclatasvir) before HCT and two other patients received Sofosbuvir-Daclatasvir (Table 3) after HCT. These two patients received DAA two and five years after HCT, respectively. Patients who received HCT DAA-based regimens had no grade 3 or 4 adverse events or antiviral discontinuation. Among all four patients who received DAA, none developed anemia, neutropenia, or thrombocytopenia while receiving anti-HCV treatment with DAA. The discontinuation rate due to adverse events was zero for IFN-free regimens. Sustained virologic responses were observed in 75% of the DAA treated patients (Table 3).

**Table 3:** - Characteristics of HCV therapy in the Hematopoietic Stem Cell Transplant recipients.

HCV therapy characteristics	IFN based therapy* 4 (100%)	DAA based therapy** 4 (100%)
HCV therapy pre HCT	4 (100)	2 (50)
Adverse events		
No	0	4 (100)
Yes	4 (100)	0
Anemia	3 (75)	0
Neutropenia	3 (75)	0
Thrombocytopenia	1 (25)	0
Flu-like syndrome	1 (25)	0
Clinical intervention due AE		
No	1 (25)	0
Yes	3 (75)	0
Ribavirin dose reduction	2 (50)	0
Erythropoietin use	1 (25)	0
Filgrastim use	2 (50)	0
Blood transfusion	2 (50)	0
HCV therapy discontinuation	1 (25)	0
Virologic response		
Recurrence	1 (25)	1 (25)
Non responder	3 (75)	0
Sustained virologic response	0	3 (75)

IFN = interferon; DAA = sofosbuvir + daclatasvir or simeprevir; AE = adverse events; *treatments from 2005 – 2012; **treatments from 2016.

### Overall outcome and mortality

All seropositive patients underwent follow-up for a median of three years after transplantation and/or HCV treatment with DAA. Among the viremic patients, most had moderate to severe liver fibrosis and had been treated for HCV at least twice. Among the HCV viremic patients, two developed acute liver disfunction immediately after transplantation. One of the patients developed CMV-associated liver dysfunction after autologous transplantation and before HCV treatment. The other patient, with liver cirrhosis, presented sinusoidal obstruction syndrome after allogeneic transplantation without medical record of subsequent HCV antiviral therapy due to loss to follow-up. The median time after transplantation to liver dysfunction for both patients was four weeks. All patients had their acute episode of liver dysfunction resolved with no further complications. No other clinical complications related to liver disease or base disease were observed during the investigation and the mortality rate during the time of observation was zero.

## DISCUSSION

In a 10-year period from January 2010 to January 2020, among 1,293 HCT recipients, seven (0.54%) were positive for anti-HCV antibodies, five (0.39%) of whom were also viremic for HCV-RNA. Most viremic patients had moderate to severe liver fibrosis and had clinical complications. Most patients had received at least two courses of HCV treatment. Administration of the new DAA was more successful than previous IFN-based treatments. No other clinical complications related to liver disease or base disease were observed during a three-year follow-up period and the mortality rate was zero.

The HCV infection has been a major concern among HCT recipients due to its high prevalence and association with several clinical complications^3^. In the past, blood transfusion was the main risk factor associated with the acquisition of HCV in this group of patients worldwide^4^. Also, in Brazil, blood transfusions have been one of the major risk factors for HCV transmission for decades. However, mandatory HCV serological screening and HCV nucleic acid detection in all samples obtained from donors in blood banks, from 1993 and 2014 respectively, has markedly decreased HCV transmission by blood transfusion in Brazil. The risk of HCV transmission by blood transfusions and from blood products has decreased enormously during the last 20 years, particularly in the last 10 years^21^. Data from the national hemovigilance report indicate that an annual average of 7.2 cases of blood-borne diseases were reported from 2007 to 2015, with an average cumulative rate of 0.20 occurrences per 100,000 transfusions, and that only 13 cases of HCV transmission following supportive hemotherapy were documented from 1995 to 2015^22^. At the same time, Brazilian blood banks have observed a decrease in the prevalence of transfusion-transmissible infections among donors, mainly HCV, likely attributed to the improvement in clinical and laboratory screening of hemotherapy services, as well as to changes in regional demographic composition. This ultimately reflects a decrease in the frequency of this infection in the community and, consequently, a reduction in the risk of blood transmission^23-29^. Our data indicating that HCV seroprevalence among HCT recipients is comparable to that of the, confirms the high quality and increased safety of blood transfusion in Brazil.

Since hemovigilance procedures related to HCV in hemotherapy services was established in 1992, a progressive, over time decrease in the prevalence of HCV infection among HST recipients could be observed worldwide. In the post-screening era of blood donors, more precisely between 1995 and 2016, HCV seroprevalence among HCT recipients declined significantly, ranging on average from 1.08% to 4.1%, with estimated viremia of 0.9% to 6%^6,8,13,30-39^. Even in Egypt, where the frequency of HCV is quite high in the general population, this epidemiological shift can also be observed^9,40^.

The HCV treatment has changed tremendously in the last 10 years^3,17^. Until recently, treatment for HCV infection was inefficient and involved IFN-based therapy. This regimen was associated with poor tolerability and low efficacy, particularly among HCT recipients. It was also associated with significant side effects and a high rate of treatment discontinuation.

Our data, showing poor tolerability and low efficacy for IFN-based therapy for HCT recipients, agrees with these prior observations. Among the five viremic patients included in our study, four received IFN-based therapy during clinical follow-up. None of them achieved a sustained anti-virologic response and each presented with grade 3 adverse events, including one case of antiviral discontinuation (grade 4 AE). Conversely, when the same four patients subsequently received DAA-based therapy during follow-up, no grade 3 or 4 adverse events were reported and most of them (three out of four patients) obtained a sustained anti-virologic response.

Immediately after transplantation, among four HCV viremic patients, two (50%) developed acute liver disfunction. The median time after transplantation to liver dysfunction was 4 weeks and all patients had their acute liver dysfunction resolved with no further complications. On a long-term basis, after at least 3 years of follow-up, no other clinical complications related to liver disease or base disease were observed and mortality was zero. Our study has some limitations that must be acknowledged. A retrospective observational study necessarily limits data acquisition to findings contained in the medical record, which may be incomplete. In addition, this investigation used transplant recipients from only a single reference center. Thus, the observed findings may not be comparable to data from other centers in Brazil or elsewhere. A major limitation is that only a small number of HCV-infected transplant patients were identified. The study, therefore, must be defined as exploratory and larger investigations should be performed to validate our findings.

## CONCLUSION

Despite the study limitations, we believe the information described in this study is valuable as it highlighting that HCV infection still occurs in a small fraction of patients undergoing HCT. Furthermore, our preliminary finding that HCV-infected HCT recipients can be successfully treated with DAA reinforces the value of testing all transplant recipients for HCV both prior to and following this procedure.
